# The Role of Pre-Operative Biopsy in Malignant Peripheral Nerve Sheath Tumours: A Review and Retrospective Series with a Management Algorithm from a Single-Center Experience

**DOI:** 10.3390/neurolint17090132

**Published:** 2025-08-22

**Authors:** Francesca Vincitorio, Leonardo Bradaschia, Enrico Lo Bue, Alice Antico, Paolo Titolo, Bruno Battiston, Diego Garbossa, Fabio Cofano

**Affiliations:** 1 Neurosurgery Unit, Department of Neuroscience “Rita Levi Montalcini”, CTO Hospital, A.O.U Città della Salute e della Scienza di Torino, University of Turin, 10126 Turin, Italy; vincitorio.francesca@gmail.com (F.V.);; 2 Hand and Upper Limb Surgery Unit, Department of Orthopaedic and Traumatology, Orthopaedic and Trauma Center, Molinette Hospital, Città della Salute e della Scienza, 10126 Turin, Italy; titolopaolo@gmail.com (P.T.);

**Keywords:** MPNST, peripheral nerve tumour, preoperative biopsy, neoadjuvant therapy, early diagnosis

## Abstract

**Background/Objectives**: Peripheral nerve tumours are commonly encountered in clinical practice. Although most are benign, a subset can exhibit aggressive and invasive behaviour, evolving into malignant peripheral nerve sheath tumours (MPNSTs). Due to their rarity and overlapping features with benign lesions, MPNSTs are frequently misdiagnosed during the initial evaluation. Preoperative biopsy may aid in distinguishing malignant from benign lesions. This single-center study aimed to develop and validate a diagnostic algorithm—based on a systematic literature review and institutional case series—to assess the role of preoperative biopsy in the diagnostic workflow. **Methods**: A systematic review of the literature was conducted in accordance with PRISMA (Preferred Reporting Items for Systematic Reviews and Meta-Analyses) guidelines, covering the period from 1998 to 2024. Additionally, a retrospective case series of patients with peripheral nerve lesions treated at the authors’ institution between January 2018 and June 2024 was analysed. **Results**: Forty-eight articles met the inclusion criteria and were categorized into five key domains: radiological features of MPNSTs, associated risk factors and genetic conditions, the role of preoperative biopsy, use of radiotherapy, and general clinical management strategies. The proposed diagnostic algorithm was applied to a series of 36 patients, four of whom met the criteria for preoperative biopsy. In three of these cases, early diagnosis of MPNSTs was achieved. **Conclusions**: Preoperative biopsy appears to be a safe and cost-effective tool for the early identification of MPNSTs. Early diagnosis may facilitate the use of neoadjuvant therapies—such as radiotherapy or chemotherapy—potentially enabling more radical surgical resection and improving overall patient outcomes.

## 1. Introduction

Peripheral nerve tumours (PNTs) are among the most common types of lesions encountered in daily clinical practice. Peripheral nerves can be affected by both primary and metastatic neoplasms [1]. Regarding primary tumours, PNTs include low malignant potential lesions such as Benign Peripheral Nerve Sheath Tumours (BPNSTs), as well as highly malignant lesions like neuroblastoma and Malignant Peripheral Nerve Sheath Tumours (MPNSTs). BPNSTs are the most common benign soft tissue tumours in the general population, with an estimated incidence of 10–12% [2]. On the other hand, MPNSTs are rare in the general population, accounting for only 2% of all sarcomas [3], and they can occur sporadically or be associated with syndromes such as Neurofibromatosis type 1 (NF1, also known as Von Recklinghausen disease) [4,5,6].

To date, the therapeutic gold standard—and the only potentially curative treatment—for malignant lesions is considered to be complete R0 surgical excision, often followed by radiation and systemic therapy, although there is no unanimous consensus on this approach [7,8,9].

Given the low incidence and prevalence of MPNSTs compared to the high frequency of BPNSTs, newly diagnosed PNTs may be underestimated during daily clinical practice and mistakenly treated with standard excisional surgery rather than a more appropriate aggressive resection. When the pathological examination reveals the malignant nature of the lesion, this diagnostic delay often results in a postponement of effective management, including adjuvant therapy.

Therefore, the aim of this study is to propose a management algorithm for the treatment of malignant PNTs and to validate it through the presented case series, with a specific focus on evaluating the overall role of pre-operative biopsy.

## 2. Materials and Methods

This systematic review was conducted in accordance with the Preferred Reporting Items for Systematic Reviews and Meta-Analyses (PRISMA) guidelines [10]. All full-text, English-language manuscripts related to the management of Malignant Peripheral Nerve Sheath Tumours (MPNSTs) and their diagnosis through various imaging modalities were screened using the PubMed/MEDLINE, Embase, Cochrane Library, Scopus, and Web of Science databases, covering the period from 1998 to 2024.

A combination of keywords was used, including: “Malignant Peripheral Nerve Sheath Tumour” OR “MPNST” AND “Management” OR “Radiotherapy” OR “Imaging” OR “PET” OR “Chemotherapy” OR “Diagnosis” OR “Neurofibromatosis” OR “Biopsy”. The objective of the search was to identify all articles related to MPNST management and/or imaging-based diagnosis.

Due to the heterogeneity and incomplete reporting of diagnostic pathways in the available literature, particularly regarding preoperative imaging, biopsy indication, and histological confirmation, a pooled analysis of literature cases was not feasible. Therefore, the algorithm developed through systematic literature synthesis was applied to a single-institution retrospective cohort, where complete clinical, radiological, and pathological data were available for all patients.

### 2.1. Study Selection

The retrieved articles were screened based on title, abstract, and full text to assess their eligibility. Discrepancies in selection were resolved by consensus.

The inclusion criteria were:

- Articles written in English;
- Case series, randomized controlled trials, and/or observational cohort studies;
- Studies specifically referring to the management of MPNSTs.

### 2.2. Outcomes

**Figure 1:**
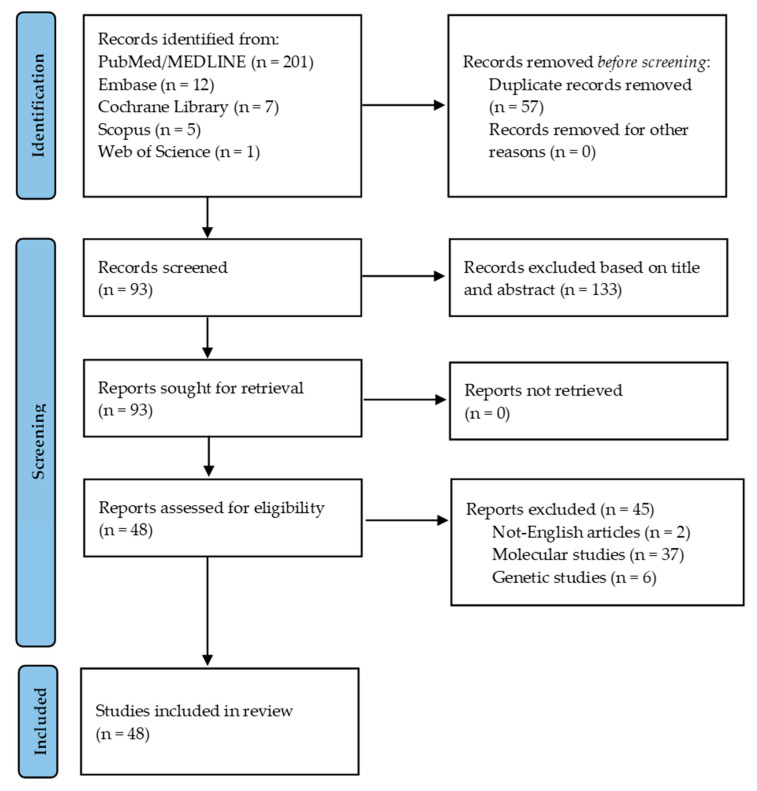
Flowchart of the systematic review. A total of 48 articles were included to formulate the algorithm. Source: https://www.bmj.com/content/372/bmj.n71, accessed on 23 July 2025.

A total of 226 manuscripts were identified in the initial search. A total of 133 articles were excluded based on title and abstract. A total 8 articles were excluded due to the unavailability of the full text or because they were not written in English. A total of 37 articles were excluded for covering unrelated topics. Ultimately, 48 articles met the inclusion criteria. A PRISMA flowchart is provided in Figure 1, and the list of included articles is summarized in Table 1.

The collected articles were categorized into five main groups based on their primary focus:

1. Radiological appearances;
2. Risk factors and/or genetic syndromes;
3. Use of pre-operative biopsy;
4. Use of radiotherapy;
5. General clinical management.

Specifically, 17 articles were assigned to the first group, 5 to the second, 5 to the third, 3 to the fourth, and 18 to the fifth. These thematic categories formed the foundation for the development of the proposed management algorithm (Table 1).

### 2.3. Case Series

A case series was conducted at the authors’ institution between January 2018 and June 2024. Inclusion criteria:

- Age ≥ 18 years;
- Clinical history of a peripheral nerve tumour (PNT) treated surgically.

Exclusion criteria:

- Lesions involving cranial nerves;
- Lesions with an intradural location (cervical, thoracic, or lumbar spine).

Personal, clinical, radiological, and histopathological data were collected, including:

- Age and gender;
- Presence of genetic disorders;
- Clinical symptoms;
- Imaging features (MRI, CT, PET, ultrasound);
- Histological diagnosis;
- Management details during hospitalization.

Although pediatric tumors such as neuroblastomas are mentioned in the introduction for contextual purposes, the present study exclusively included patients aged over 18 years. This choice was made to examine and validate the proposed management algorithm specifically within an adult population, ensuring greater homogeneity in terms of clinical presentation, decision-making processes, and follow-up protocols.

This retrospective study analysed anonymized clinical data collected as part of routine patient care. Specific informed consent for inclusion in this study was not required according to institutional policies, and Institutional Review Board (IRB) approval was therefore not sought. Nevertheless, all patients provided informed consent for the surgical procedures and diagnostic interventions they underwent. This study was conducted in accordance with the Declaration of Helsinki and the STROBE guidelines for observational studies.

### 2.4. Histological Diagnosis

The histopathological diagnosis was established by board-certified neuropathologists with expertise in peripheral nerve sheath tumours. The tumours showed the typical features of a high-grade spindle cell neoplasm, including hypercellularity, nuclear atypia, brisk mitotic activity, and areas of necrosis. Although no pathognomonic immunohistochemical markers exist for malignant peripheral nerve sheath tumours (MPNSTs), the diagnosis was supported by positive staining for SOX10 and S100, the absence of markers indicative of other spindle cell malignancies (e.g., cytokeratins, desmin, CD34), and a consistent clinical and radiological context. Final diagnosis was rendered through an integrated assessment of histological, immunophenotypic, and clinical features, in accordance with the 2020 WHO Classification of Soft Tissue and Bone Tumours.

## 3. Results

### 3.1. Algorithm

**Figure 2:**
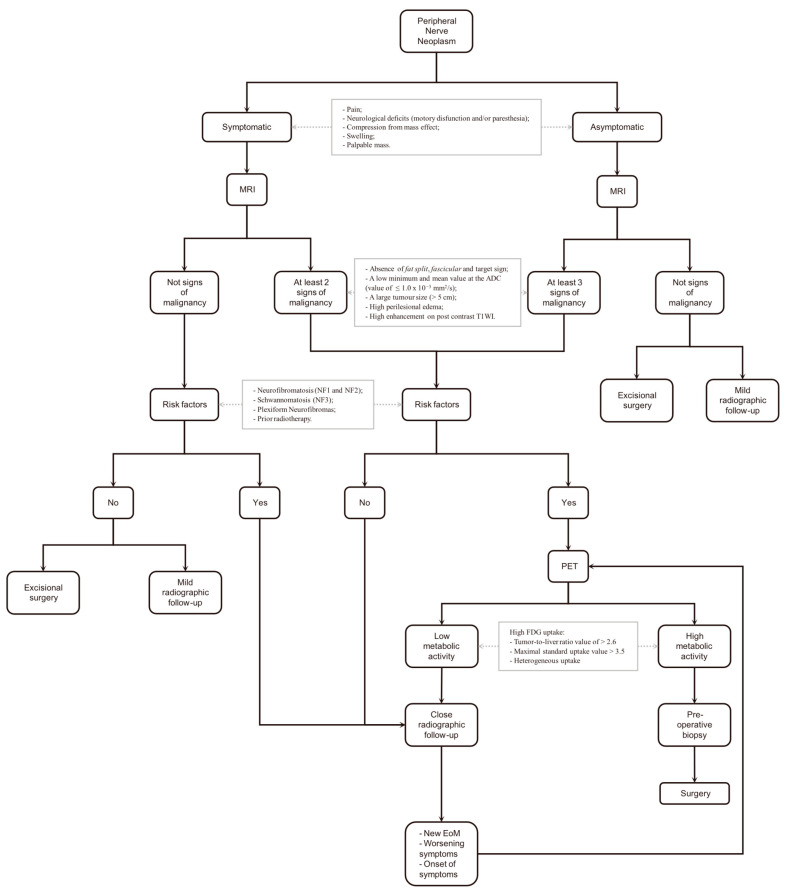
The proposed algorithm. Its explanation might be found in the text. MRI: Magnetic Resonance Imaging; ADC: Apparent Diffusion Coefficient; WI: weighted imaging; NF: neurofibromatosis; FDG: fluorodeoxyglucose; PET: Positron Emission Tomography; EoM: Evidence of Malignancy.

Based on the data collected from the systematic review, a management algorithm was developed for newly diagnosed peripheral nerve tumours (PNTs) (Figure 2).

The first step is the clinical evaluation: a symptomatic lesion should be identified through symptoms such as pain; neurological deficits (motor dysfunction and/or paraesthesia); and mass effect causing compression of nearby structures, swelling, or the presence of a palpable mass.

The second step involves contrast-enhanced MRI as the initial imaging diagnostic tool. Four different scenarios may arise from the MRI findings:

An asymptomatic lesion without evidence of malignancy (EoM) is reasonably considered a common benign peripheral nerve sheath tumour (BPNST), and management options include excisional surgery or mild radiographic follow-up.

A symptomatic lesion without EoM should be considered potentially malignant, albeit with low probability. If no risk factors are present, either excisional surgery or mild radiographic follow-up can be proposed. However, if risk factors exist, a closer radiographic follow-up is recommended.

A symptomatic lesion with at least two EoMs on MRI, or an asymptomatic lesion with at least three EoMs on MRI, both without associated risk factors, are considered to have a higher potential for malignancy and should be further evaluated with an ^18^F-FDG PET/CT scan.

Evidence of Malignancy (EoM) is defined as:

- Absence of fat split, fascicular, and target signs;
- Low minimum and mean ADC values (≤1.0 × 10^−3^ mm^2^/s);
- Large tumour size (>5 cm);
- High perilesional edema;
- High enhancement on post-contrast T1-weighted images (T1WI).

Risk factors include:

- Neurofibromatosis types 1 and 2 (NF1 and NF2);
- Schwannomatosis (NF3);
- Plexiform neurofibromas;
- Prior radiotherapy (regardless of indication).

Regarding follow-up timing, a new contrast-enhanced MRI is recommended after one month for close radiographic follow-up and after three months for mild radiographic follow-up.

If new EoMs appear or previous findings worsen on follow-up imaging, or if symptoms develop in a previously asymptomatic patient or worsen during outpatient visits, an ^18^F-FDG PET/CT is suggested.

^18^F-FDG PET/CT is recommended to exclude a high-metabolic-rate neoplasm. High metabolic FDG uptake is defined as:

- Tumour-to-liver ratio > 2.6;
- Maximal standardized uptake value (SUVmax) > 3.5;
- Heterogeneous uptake pattern.

A lesion suspected to have a high metabolic rate warrants a pre-operative, image-guided, core-needle biopsy for definitive characterization. Surgical planning will then depend on the histopathological diagnosis: neoadjuvant chemo/radiotherapy followed by radical surgery for aggressive lesions, or simple excisional surgery for benign tumours.

If low metabolic activity is demonstrated, close follow-up is still recommended, following the previous guidelines.

In conclusion, pre-operative biopsy is generally avoided in cases with clinical and radiological features strongly suggestive of benign peripheral nerve sheath tumours, primarily due to the risk of neurological injury. In our proposed algorithm, PET-CT serves as a discriminative tool to identify cases where malignant transformation is suspected, thus guiding the selective use of tissue diagnosis.

A total of 69 patients were initially evaluated at our institution: 15/69 (21.7%) had acoustic neuromas, 3/69 (4.5%) cervical spinal schwannomas, 3/69 (4.5%) dorsal spinal schwannomas, and 12/69 (17.4%) lumbar spinal schwannomas. Subsequently, 33 patients were excluded based on the exclusion criteria. The remaining 36 patients were enrolled to test the validity of the proposed algorithm.

Of these, 17/36 patients (47.2%) were asymptomatic and showed no evidence of malignancy (EoM) on contrast-enhanced MRI, except for one patient. All except this patient were managed as BPNSTs with standard excisional surgery. Histopathological analysis revealed 15/36 cases (41.7%) with Schwannomas or ancient Schwannomas (WHO grade I) and 1/36 case (2.8%) with Glomus tumour (a mesenchymal tumour) (Table 2). The single patient with EoM was later diagnosed with Neurofibromatosis type 1 (NF1) and underwent ^18^F-FDG PET/CT and a pre-operative CT-guided biopsy, which confirmed a low-grade MPNST. Gross total resection (GTR) was performed (Case 3).

The remaining 19/36 patients (52.8%) presented with symptoms related to the neoplasm. Among these, 5/36 (13.9%) demonstrated at least two EoMs on imaging. Of the remaining 14/36 (38.9%), excisional surgery was offered due to the absence of risk factors, with final histopathological diagnoses showing Schwannomas or ancient Schwannomas (WHO grade I). Among the 5 patients with EoM on imaging, 2/5 had NF1 syndrome, 1/5 had undergone prior radiotherapy for head and neck cancer, and 2/5 had no identifiable risk factors. The latter two patients underwent follow-up MRI at one month, which showed no changes; they then underwent conventional excisional surgery revealing a Ganglioneuroma (2.8%) and a Haemangioma (2.8%), respectively.

An ^18^F-FDG PET/CT was performed on the remaining patients, showing increased metabolic activity in 2 out of 3 patients with suspected neoplasms. The single patient with intermediate-low FDG uptake repeated an MRI after one month, which showed slight worsening of the EoM, leading to biopsy. Regardless of the biopsy technique used (US/CT-guided or open biopsy), histopathology revealed early diagnosis of MPNST in 2/3 cases and a fibromatosis-desmoid lesion (a locally aggressive fibroblastic tumour) in the remaining case. All patients underwent surgery; gross total resection (GTR) was achieved in one MPNST patient (Case 1) and in the fibromatosis-desmoid case, while subtotal resection (STR) was performed in the other MPNST patient due to anatomical constraints (Case 2).

The patient with desmoid-type fibromatosis presented with a rapidly growing soft tissue mass clinically and radiologically suggestive of MPNST. A percutaneous biopsy confirmed the diagnosis of fibromatosis, and GTR was subsequently performed. This case underscores the importance of obtaining histological confirmation before proceeding to definitive surgery, especially in borderline lesions, and may represent a scenario where immediate resection without biopsy might have led to overtreatment or an unnecessarily aggressive surgical approach. We discuss this further below.

Neoadjuvant radiotherapy was proposed in one case only, as was chemo/radiotherapy post-surgery (Table 3).

At the histological examination, all patients had no specified type of MPNST.

Case reports of the above-mentioned MPNST cases are described below.

### 3.2. Case 1

**Figure 3:**
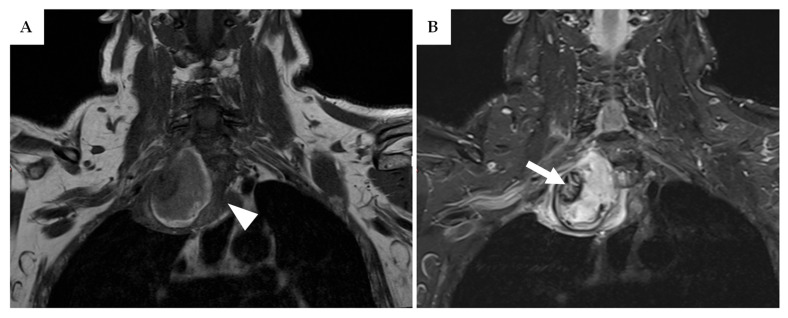
(**A**) T1-weighted TSE MRI of patient 1, showing a coronal view of the suspected lesion involving the right brachial plexus. The presence of extensive perilesional edema (white arrowhead) and a large tumour diameter of 70 mm in a patient with NF1 is highly suggestive of malignant transformation of a plexiform neurofibroma (PN). (**B**) T2-weighted STIR MRI of the same coronal slice as in image (**A**). A T1- and T2-hypointense nodule (white arrow) is visible in the mid-right portion of the tumour, which was later identified as a necrotic focus indicative of malignant transformation.

In March 2019, a 49-year-old man with a diagnosis of Neurofibromatosis type 1 (NF1) since age 10 presented with a tingling sensation in his right forearm persisting for a couple of months. An MRI performed the previous year had revealed a neurofibroma on the right brachial plexus measuring approximately 60 mm in diameter. Over the past week, the paraesthesia had progressed to burning pain. A follow-up contrast-enhanced MRI after one month showed a slight enlargement of the soft tissue mass (70 mm vs. 60 mm), which was heterogeneously enhancing, T1 hypointense, and T2 hyperintense, with high perilesional edema and absence of the fat split sign (Figure 3). Given his genetic condition, the patient underwent an ^18^F-FDG PET/CT scan, which demonstrated high metabolic activity localized to a 10 mm area at the lesion’s centre. Due to the lesion’s size and depth, an open pre-operative biopsy was performed, strongly suggesting malignancy. Consequently, an extensive gross total resection (GTR) was carried out instead of classical excision. Final histopathology confirmed a malignant peripheral nerve sheath tumour (MPNST), and adjuvant radiotherapy was administered. The patient is currently alive and under follow-up. At the last outpatient visit in 2023, he continued to report forearm paraesthesia, mild hypoesthesia of the hand, and a slight global upper limb strength deficit graded 3/5 on the Medical Research Council (MRC) scale.

### 3.3. Case 2

**Figure 4:**
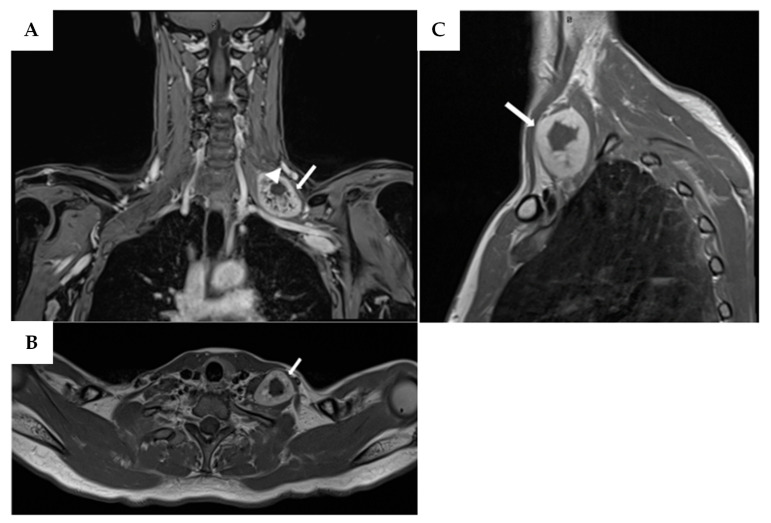
Enhanced T1-weighted-TSE MRI of patient 2 showing in the three axes the neoplasm at the base of the neck on the left brachial plexus. (**A**) Coronal view, the mass (white arrow) is located just above the subclavian artery and shows a central area of necrosis (arrowhead). (**B**) Axial view, showing the relationship of the mass with the scalene muscles. (**C**) The sagittal view reveals the close position in respect to the collar bone and the sternocleidomastoid muscle.

In November 2021, a 46-year-old woman presented to the emergency room with neck pain and left upper limb weakness ongoing for two weeks. Neurological examination revealed muscle strength graded 3/5 on the MRC scale in the left biceps and triceps, and 2/5 distally in the left upper limb. Her medical history was significant due to childhood mantle field radiation therapy for Hodgkin’s lymphoma, from which she had fully recovered. Given the typical extent of mantle field radiation, it is likely that the left brachial plexus lay within the radiation field. At the time of presentation, she was in long-term remission and not receiving any lymphoma-related treatment, nor was she under immunosuppressive therapy. Ultrasound revealed a subcutaneous lesion measuring approximately 45 mm. Subsequent contrast-enhanced MRI identified multiple peripheral nerve-related lesions, the largest measuring 52 mm, located on the left brachial plexus (Figure 4). An ^18^F-FDG PET/CT scan showed peripheral increased metabolic activity in the lesion. An ultrasound-guided biopsy was performed, which diagnosed early MPNST. Due to complex anatomy, a subtotal resection (STR) was carried out. Histopathological examination confirmed the biopsy diagnosis. The patient underwent adjuvant chemotherapy with doxorubicin and radiation therapy. No postoperative strength deficits were reported. At one-year follow-up MRI, local recurrence and pulmonary metastases were identified. The patient passed away a few months later due to a lung infection.

### 3.4. Case 3

**Figure 5:**
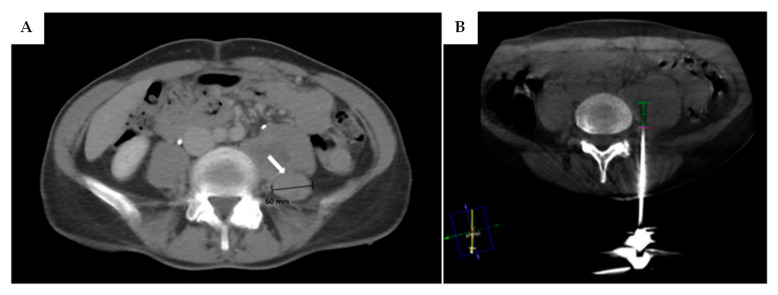
(**A**) CT scan of the low abdomen of patient 3 showing the round neoplasm (white arrow) located behind the left psoas muscle at L4-L5 level, measuring ~60 mm in diameter. (**B**) CT-guided biopsy was performed to rule out the hypothesis of a malignant lesion, which it was later confirmed to be.

In early 2023, a 51-year-old woman was admitted to the emergency room following a fall at home, resulting in a sacral fracture. The fracture did not require surgical intervention, but a CT scan incidentally revealed a round mass posterior to the left psoas muscle at the L4-L5 level. The patient reported a family history of NF1 (her mother), although she had never undergone genetic testing. She was discharged with instructions for MRI and genetic counselling. Three months later, outpatient genetic testing confirmed NF1, despite absence of cutaneous stigmata. Contrast-enhanced MRI characterized the lesion as a suspected schwannoma of the lumbar plexus, measuring 60 mm × 30 mm, showing strong post-contrast enhancement and very low minimum and average ADC values. Surgery was proposed, but an ^18^F-FDG PET scan was performed first, revealing high FDG uptake. A CT-guided biopsy (Figure 5) confirmed low-grade MPNST. Neoadjuvant radiotherapy was administered, resulting in significant tumour volume reduction (45 mm vs. 60 mm). Gross total resection was then performed, including removal of part of the psoas muscle and surrounding fatty tissue. Final histopathology confirmed the diagnosis. The patient continued adjuvant radiotherapy. At the 3-month postoperative follow-up, MRI showed no recurrence, and the patient had minimal left thigh flexion weakness but no paraesthesia or dysesthesia.

## 4. Discussion

While benign peripheral nerve sheath tumours (BPNSTs) represent the most common form of peripheral nerve tumours, malignant peripheral nerve sheath tumours (MPNSTs) are relatively rare, accounting for approximately 2% of all soft tissue sarcomas. Sarcomas themselves are an uncommon group of malignancies, with a global incidence estimated at around 5 cases per million of the population annually [3]. This rarity significantly hampers large-scale studies and contributes to the limited evidence regarding optimal diagnostic strategies, treatment approaches, and prognosis.

Although the exact cellular origin of MPNSTs remains uncertain, the majority of cases arise in association with peripheral nerves, suggesting a derivation from Schwann cells or their precursors [41]. Alternative hypotheses point to neural crest-derived stem cells as a possible origin, but further research is required to fully elucidate the underlying mechanisms of tumorigenesis. MPNSTs may occur sporadically or in association with neurofibromatosis type 1 (NF1), a genetic disorder that markedly increases the lifetime risk of developing these tumours. In NF1 patients, MPNSTs may develop de novo, from malignant transformation of a pre-existing neurofibroma, or—more rarely—from a schwannoma [52]. This spectrum reflects the biological heterogeneity of these tumours and explains their varied clinical presentations.

In addition to NF1, known risk factors for MPNST include a positive family history and prior exposure to ionizing radiation. Radiation-induced MPNSTs account for approximately 3–10% of all cases and are often associated with a more aggressive clinical course and poorer prognosis compared to sporadic or NF1-associated forms [4]. These findings underscore the importance of long-term surveillance in patients with a history of therapeutic irradiation, particularly involving the head and neck region.

Radiological evaluation, particularly magnetic resonance imaging (MRI), plays a pivotal role in the assessment of peripheral nerve tumours. Certain imaging features—such as rapid growth, ill-defined margins, perilesional edema, and heterogeneity—can suggest malignant transformation. In NF1 patients, large plexiform neurofibromas, atypical nodular lesions, and distinct signal changes may raise suspicion for MPNST [28]. Advanced techniques such as diffusion-weighted imaging and positron emission tomography (PET) with ^18^F-FDG are increasingly used to improve diagnostic accuracy, although their routine application remains to be standardized.

From an epidemiological perspective, the median age at diagnosis of sporadic MPNST is approximately 41 years, while NF1-associated cases tend to present earlier, often during the third decade of life [53]. Clinically, MPNSTs typically present as painful, firm, and rapidly enlarging masses along peripheral nerves, frequently accompanied by neurological deficits such as sensory loss or motor weakness [7,49].

The mainstay of treatment for localized MPNST remains complete surgical excision with negative margins (R0 resection), as this is currently the only potentially curative option. The literature does not report significant differences in the surgical approach between low-grade and high-grade MPNST.

Adjuvant radiotherapy is frequently used to improve local control, although its effect on overall survival remains controversial [9]. It is typically recommended for high-grade lesions or tumours larger than 5 cm [27]. Similarly, systemic chemotherapy has demonstrated limited benefit in most studies but may be considered in high-risk or metastatic cases. Ongoing research into targeted therapies and molecularly guided treatments holds promise for improved outcomes in selected patient populations.

In cases of metastatic disease, management generally follows soft tissue sarcoma protocols and includes systemic chemotherapy. However, due to the intrinsic resistance of MPNSTs to conventional agents and the overall poor prognosis in advanced stages, treatment often shifts toward palliative strategies aimed at symptom control and quality of life [46]. Recent advances in genomic profiling and immunotherapeutic approaches are promising but still require validation in larger clinical trials.

In our case series, 3 out of 36 patients were ultimately diagnosed with MPNST. Given the small sample size, a formal statistical comparison with published cohorts was not feasible. However, we performed a qualitative assessment of key clinical and demographic variables, which appear consistent with data from the literature. The mean age at diagnosis in our cohort was 48.7 years, aligning with findings reported by Arshi et al., 2015 [54]. Two of our patients had neurofibromatosis type 1 (NF1), while the third developed MPNST following childhood radiotherapy to the head and neck region for pediatric lymphoma—both recognized risk factors for MPNST.

Interestingly, another NF1 patient in our series, who presented with a symptomatic lesion and evidence of edema on MRI, was ultimately diagnosed with a desmoid-type fibromatosis. Although not classically associated with NF1, this tumour showed intense ^18^F-FDG uptake on PET imaging, illustrating the diagnostic overlap between benign and malignant peripheral nerve sheath tumours, particularly in patients with a genetic predisposition.

Regarding tumour localization, 33.3% of MPNSTs in our cohort were located in the proximal lower limb and 66.7% in the proximal upper limb. These distributions are consistent with data from Sobczuk et al., 2022 [55], who reported a predilection for the trunk and proximal limb segments. While our limited follow-up (maximum 72 months) and small sample preclude definitive conclusions regarding survival, we note that one patient died 17 months after diagnosis. The literature suggests that surgical resection remains a critical prognostic factor, as highlighted by the 5-year survival rate of 61.9% and median overall survival of 126.5 months reported by Sobczuk et al. [55].

Taken together, these findings support the clinical relevance of our proposed management algorithm, particularly the central role of preoperative biopsy in establishing a definitive histological diagnosis and guiding optimal treatment.

Given the diagnostic challenges in differentiating BPNSTs from MPNSTs preoperatively, the management of peripheral nerve tumours remains a subject of ongoing debate. In clinical practice, therapeutic decisions are often made on a case-by-case basis, reflecting the lack of standardized protocols. The algorithm proposed in this study aims to address this gap by integrating clinical, radiological, and pathological data to support risk stratification and guide the use of preoperative biopsy. Designed for practical application, it begins at the time of initial identification—either incidental or symptomatic—of a peripheral nerve tumour and offers a structured approach for managing this complex and heterogeneous group of neoplasms.

Future research should prioritize the identification of reliable biomarkers and standardized imaging criteria to improve diagnostic accuracy. Moreover, the development of targeted therapies based on molecular tumour profiling may offer new therapeutic avenues for patients with unresectable or metastatic MPNST. Ultimately, a multidisciplinary approach—encompassing neurosurgery, oncology, pathology, and radiology—is essential to optimize outcomes in this challenging clinical entity.

### 4.1. Symptoms

Symptoms play a fundamental role in the clinical assessment of any pathology, particularly during outpatient evaluations. Benign peripheral nerve sheath tumours, such as schwannomas, are often asymptomatic or present with mild symptoms [56]. These may include a foreign body sensation—especially in superficial lesions—moderate localized pain, a positive Tinel sign, or numbness and paraesthesia in the distribution of the affected nerve. While such symptoms are frequently mild, they can also overlap with those observed in more aggressive lesions, albeit with increased severity.

In malignant peripheral nerve sheath tumours, symptomatology tends to be more pronounced and debilitating [5]. Patients often report burning dysesthesia, intense and persistent pain, unexplained weight loss, and motor neurological deficits. Additionally, cosmetic deformities resulting from tumour growth and symptoms related to the compression of adjacent anatomical structures may be present. These clinical features should be considered red flags that raise suspicion for malignancy [31]. However, it is important to emphasize that no pathognomonic clinical sign definitively distinguishes benign from malignant peripheral nerve tumours. Large benign soft tissue tumours can sometimes produce symptoms indistinguishable from those of MPNSTs, complicating the clinical picture.

Therefore, while thorough clinical evaluation remains a critical component of patient assessment, it is insufficient on its own to dictate the final management strategy. Imaging studies, starting with first-level modalities such as ultrasound and MRI, are mandatory to characterize the lesion more accurately. MRI, in particular, provides detailed anatomical and tissue characterization, aiding in the identification of features suggestive of malignancy, such as irregular margins, heterogeneity, necrosis, and perilesional edema.

### 4.2. Radiological Appearance on MRI

As with all soft tissue lesions, magnetic resonance imaging remains the gold standard for evaluating suspected BPNSTs. MRI not only characterizes the neurogenic lesion but also delineates its anatomical relationships with adjacent structures, which is crucial for surgical planning.

BPNSTs typically exhibit a fusiform shape with tapered ends and several characteristic imaging signs. The “split-fat” sign, represented by a thin rim of fat surrounding the lesion on T1-weighted images, and the “fascicular” sign, consisting of multiple small ring-like structures with peripheral hyperintensity corresponding to fascicular bundles on T2-weighted images, are frequently observed [2,12,14]. These lesions generally demonstrate low-to-intermediate signal intensity on T1-weighted images and homogeneously high signal intensity on T2-weighted images, often accompanied by a central hypointense area on diffusion-weighted imaging (DWI) [2,12,14]. On contrast-enhanced T1-weighted sequences, BPNSTs usually present with central enhancement [17], and typically do not show restricted diffusion, as indicated by apparent diffusion coefficient (ADC) values greater than 1.0 × 10^−3^ mm^2^/s [2,18,25,26]. Additionally, these tumours are often aligned longitudinally along the course of the affected nerve [12] and may display the “target” sign, characterized by peripheral myxoid material and central fibrous tissue [17,23]. Importantly, cystic changes are generally absent in BPNSTs [24]. Tumour size is another important factor; BPNSTs tend to grow slowly and are more likely benign when measuring less than 5 cm in diameter [2,11,14,17].

In contrast, malignant peripheral nerve sheath tumours (MPNSTs) exhibit more aggressive imaging features. They are typically larger than 5 cm, rapidly growing masses with infiltrative margins and associated perilesional soft tissue edema [2,11,14]. The fat at the lesion’s edges is often obliterated, resulting in the absence of the split-fat sign [12]. MPNSTs display heterogeneous signal intensity on T1-weighted images, reflecting central necrosis and hemorrhage, which contribute to intratumoral cystic changes [2,14,16,19,23]. Post-contrast images often reveal avid peripheral nodular enhancement [17]. On DWI, MPNSTs lack the target sign and demonstrate restricted diffusion with minimum ADC values ≤ 1.0 × 10^−3^ mm^2^/s [18,19,24,25,26], consistent with their higher cellularity and aggressive nature.

Despite these characteristic imaging findings, it is important to emphasize that no single radiological feature is pathognomonic for MPNSTs. Overlaps in imaging appearances between benign and malignant lesions persist, underscoring the need for a comprehensive approach combining clinical evaluation, advanced imaging, and histopathological confirmation to establish a definitive diagnosis.

### 4.3. Genetic and Other Risk Factors

Beyond MRI findings, the association with Neurofibromatosis type 1 syndrome has been consistently linked to a significantly higher risk of malignant peripheral nerve sheath tumours [27,28,29,30,31]. In patients with NF1, atypical neurofibromas are widely recognized as precursor lesions to MPNSTs, and their early identification—even in asymptomatic individuals—can be lifesaving [22]. Consequently, obtaining a comprehensive personal and family medical history to detect the presence of phacomatoses is strongly recommended during patient evaluation.

It is important to note that not only NF1 but also other related disorders such as Neurofibromatosis type 2 (NF2) and Schwannomatosis contribute to an increased risk of peripheral nerve sheath malignancies. Among the various lesion types associated with these syndromes, plexiform neurofibromas (PN) are particularly significant due to their elevated likelihood of malignant transformation [27,28,29,30,31].

Another well-established risk factor for MPNST development is prior radiation therapy, irrespective of the original indication. Epidemiological data indicate that approximately 10–13% of patients diagnosed with MPNST have a history of therapeutic irradiation [42,50]. Despite this correlation, the precise pathophysiological mechanisms underlying radiation-induced MPNST formation remain incompletely understood. Hypotheses include radiation-induced DNA damage leading to oncogenic mutations in Schwann cells or their precursors, as well as alterations in the tumour microenvironment that may facilitate malignant transformation.

Given these considerations, heightened surveillance and a multidisciplinary approach are crucial for patients with known risk factors, including those with NF1 and prior radiation exposure, to enable timely diagnosis and intervention.

### 4.4. Positron Emission Tomography

Considered a second-line imaging modality, positron emission tomography with ^18^F-labeled fluorodeoxyglucose (FDG) is a functional technique that assesses tissue metabolism by measuring glucose uptake. This approach represents a significant advancement in the evaluation of peripheral nerve sheath tumours (PNSTs), as it provides insights into tumour biology beyond what is achievable with conventional anatomical imaging.

Benign PNSTs (BPNSTs) typically exhibit absent or low FDG uptake, consistent with their limited metabolic activity. In contrast, malignant PNSTs (MPNSTs) demonstrate markedly increased and often heterogeneous FDG accumulation. Quantitative thresholds—such as a maximum standardized uptake value (SUVmax) > 3.5 or a tumour-to-liver (T/L) ratio > 2.6—have been reported as useful criteria to differentiate malignant from benign lesions [15,20,21,22].

Beyond its diagnostic role, FDG PET/CT is also valuable for staging. Approximately 30–60% of patients with newly diagnosed MPNST present with metastatic disease, most commonly involving the lungs, liver, and bones [21]. PET imaging allows for whole-body assessment, offering crucial prognostic information and guiding treatment strategy.

Importantly, ^18^FDG PET/CT is particularly informative in the context of neurofibromatosis type 1, where neurofibromas may demonstrate phenotypic mosaicism—with both benign and malignant components within the same lesion. This reflects the stepwise malignant transformation of BPNSTs into low-grade MPNSTs. In such cases, PET/CT can identify metabolically active regions within otherwise indolent-appearing tumours, allowing for targeted biopsy of suspicious areas and reducing the risk of sampling error.

In this context, ^18^FDG PET/CT not only complements conventional imaging by adding a functional dimension, but also plays a key role in biopsy planning—especially in anatomically complex or radiologically ambiguous lesions. Several studies have addressed the challenge of distinguishing benign from malignant PNSTs using multicentre pooled data. In particular, Salamon et al. demonstrated that PET/CT significantly reduced unnecessary biopsies in NF1-associated lesions, while maintaining high diagnostic sensitivity [57].

These findings directly support the rationale behind our proposed algorithm, in which PET/CT is integrated as a decisive factor to guide biopsy indication. Its inclusion allows for more selective tissue sampling, improves diagnostic accuracy, and ultimately contributes to a more tailored and evidence-based management approach.

### 4.5. Preoperative Biopsy

In the oncological setting, the definitive diagnosis of MPNSTs ultimately depends on histopathological examination. Given that radical surgical resection remains the only potentially curative treatment, obtaining a precise preoperative diagnosis through core-needle biopsy is of critical importance. This approach not only confirms malignancy but also facilitates the planning of neoadjuvant therapies—such as preoperative radiation and/or chemotherapy—aimed at reducing tumour volume and improving surgical outcomes [32,34].

Histologically, MPNSTs typically consist of a fascicular spindle cell neoplasm with characteristic features including alternating hypercellular and hypocellular areas, perivascular accentuation of tumour cells, and a stroma that may range from myxoid to fibrous in composition [33,57]. Despite these typical patterns, definitive diagnosis remains challenging due to significant cytomorphologic overlap with a variety of other benign and malignant soft tissue tumours. In this context, the application of modern immunohistochemical markers has substantially enhanced diagnostic accuracy, allowing better discrimination of MPNSTs from histologic mimics [33,34].

A percutaneous image-guided core-needle biopsy is the recommended approach to confirm or exclude MPNST following suspicious imaging findings. When the lesion is superficial or otherwise accessible, ultrasound or computed tomography (CT) guidance is preferred to ensure precise targeting and minimize complications [32,34,35,36]. However, for deeply located tumours adjacent to vital neurovascular or musculoskeletal structures, an open biopsy may be necessary to obtain adequate tissue samples safely.

An alternative approach is intraoperative frozen section analysis, a commonly available diagnostic tool; however, its utility in the diagnosis of MPNST is limited. MPNST diagnosis relies on nuanced morphological features, which may be compromised by freezing artifacts, as well as on clinical history and a limited panel of immunohistochemical markers such as S100 and SOX10. These markers lack specificity and are frequently expressed in other soft tissue tumours, and their detection often requires time-consuming techniques not compatible with intraoperative frozen section workflows. Therefore, when MPNST is suspected, preoperative biopsy remains the preferred strategy to ensure an accurate diagnosis and guide appropriate surgical planning.

It is important to balance the benefits and risks of preoperative biopsy. A major concern is the potential for nerve injury during biopsy, which could lead to neurological deficits. Additionally, sampling errors and insufficient tissue may result in false-negative findings, delaying diagnosis and treatment. For these reasons, preoperative biopsy is generally positioned at the end of the diagnostic algorithm, after thorough imaging assessment. In this context, FDG PET/CT plays a crucial role by identifying areas within the tumour with the highest metabolic activity, guiding biopsy to the most representative sites and thereby reducing false-negative rates.

The decision to perform a biopsy ultimately weighs the risk of nerve damage against the substantial benefits of an early and accurate malignant diagnosis. Early identification of MPNST enables timely neoadjuvant therapy [58,59,60,61], which can reduce tumour burden and improve the likelihood of complete surgical resection, ultimately enhancing patient survival outcomes [62].

Importantly, preoperative biopsy should be reserved only for cases considered highly suspicious for malignancy based on clinical and radiological findings. In lesions deemed likely benign, or in which suspicion remains low, it is generally appropriate to proceed directly to complete surgical excision without prior biopsy. This approach helps avoid unnecessary procedural risks, including potential nerve injury, and reduces diagnostic delays.

The case of desmoid-type fibromatosis, although not central to our series focused on MPNST, raises important considerations regarding diagnostic workup. Given its aggressive clinical and radiological appearance, it could have been mistaken for MPNST and resected upfront. However, percutaneous biopsy avoided misdiagnosis and allowed for an appropriate, margin-negative surgery. This supports the notion that biopsy remains a crucial step even when imaging suggests malignancy, particularly when the diagnosis might significantly alter surgical planning.

## 5. Conclusions

Although sampling errors can occur with both core-needle and open biopsies, as reported by Pendleton et al. [35], a low-cost needle biopsy in selected patients can expedite the diagnostic process, potentially enabling a more radical surgical approach in cases of malignant peripheral nerve sheath tumours. This approach may be further optimized by integrating neoadjuvant radiotherapy and subsequent adjuvant chemo- and/or radiotherapy. Moreover, prior PET/CT imaging not only assists in excluding lesions with low metabolic activity but also aids in targeting the most representative area for biopsy, particularly in cases where mosaicism of benign and malignant cellular components is suspected.

In conclusion, we believe that the benefits of preoperative biopsy clearly outweigh its potential risks—such as bleeding from the biopsied lesion, fibrotic scar formation that may alter future surgical anatomy, tumour seeding, and sampling errors. When performed in appropriately selected patients and integrated with advanced imaging, preoperative biopsy represents a valuable tool in the diagnostic and therapeutic management of peripheral nerve sheath tumours.

Future work should focus on prospective validation of this decision-making tool through pooled analyses or multicentre collaborations, especially to refine the role of biopsy in complex diagnostic settings.

## 6. Limitations

The proposed algorithm is primarily grounded in the currently available literature; however, certain criteria within it remain inevitably somewhat arbitrary, due to the limited consensus and paucity of robust data in this specific clinical area. For instance, the cut-off values established for the Evidence of Malignancy (EoM) and the recommended timing for radiological follow-up have been defined based on the best available evidence, but they are not yet standardized across institutions or studies. This introduces a degree of subjectivity and potential variability in the algorithm’s application and clinical outcomes.

Moreover, while PET/CT imaging provides valuable information on the metabolic activity of peripheral nerve sheath tumors, its diagnostic specificity is limited by the potential for false-positive results. Benign or inflammatory conditions may also exhibit increased FDG uptake, potentially leading to overestimation of malignancy risk. In light of this, PET/CT was deliberately positioned later in the diagnostic pathway—following initial clinical evaluation and high-resolution MRI—to improve diagnostic accuracy and minimize unnecessary invasive procedures.

Another limitation of our study is the exclusion of PET/MRI from the diagnostic algorithm. Although PET/MRI offers superior soft tissue contrast and reduces radiation exposure compared to PET/CT, it is not yet widely available in most clinical settings. Furthermore, the current body of literature specifically addressing the role of PET/MRI in the evaluation and early detection of MPNSTs is still limited, preventing its routine integration into our proposed diagnostic workflow.

A further critical limitation lies in the role of preoperative biopsy. Although histopathological assessment remains the gold standard for definitive diagnosis, obtaining a representative tissue sample from PNSTs is particularly challenging. These tumours are often heterogeneous, both morphologically and at the molecular level, which complicates accurate sampling and may result in false-negative findings. Furthermore, percutaneous or open biopsy procedures carry a non-negligible risk of damaging the involved nerve, potentially leading to new or worsened neurological deficits. This risk must be carefully balanced against the potential diagnostic benefit, particularly in lesions where imaging and clinical data already strongly suggest a benign or indolent behaviour.

Additionally, the algorithm was retrospectively validated on a relatively small and heterogeneous cohort from a single institution. This limited sample size restricts the external validity of our findings and may introduce selection and institutional biases. Although the algorithm was developed based on local data and clinical judgment, its broader applicability requires validation in larger, multicentre cohorts. Notably, pooled analyses of individual patient data have been successfully employed in the study of other rare diseases, offering enhanced statistical power and more generalizable conclusions. Therefore, prospective, multicentre studies are warranted to evaluate the clinical utility, reproducibility, and prognostic impact of our proposed approach. Such efforts would also enable refinement of the algorithm’s thresholds and parameters, fostering its adaptation to diverse healthcare settings and patient populations.

## Figures and Tables

**Table 1:** Repartition of the 48 included articles. The five main categories are radiological appearances, risk factors and/or genetic diseases, use of pre-operative biopsy, use of radiotherapy and general clinical management.

Group	N° Studies	Authors, Year	Title
Radiological appearance	17	Lang N et al., 2012 [11]	Malignant peripheral nerve sheath tumor in spine: imaging manifestations
		Gosein M et al., 2013 [12]	Plexiform neurofibroma of the wrist: imaging features and when to suspect malignancy
		Sperandio M et al., 2013 [13]	Malignant Peripheral Nerve Sheath Tumour: CT and MRI Findings
		Rafailidis V et al., 2014 [14]	Imaging of the malignant peripheral nerve sheath tumour with emphasis οn ultrasonography: correlation with MRI
		Khiewvan B et al., 2014 [15]	The value of ^18^F-FDG PET/CT in the management of malignant peripheral nerve sheath tumors
		Salamon J et al., 2015 [16]	Multimodal Imaging in Neurofibromatosis Type 1-associated Nerve Sheath Tumors
		Yu YH et al., 2016 [17]	Radiological findings of malignant peripheral nerve sheath tumor: reports of six cases and review of literature
		Karsy M et al., 2016 [18]	Diagnostic Quality of Magnetic Resonance Imaging Interpretation for Peripheral Nerve Sheath Tumors: Can Malignancy Be Determined?
		Aran S et al., 2017 [19]	Radiologic manifestation of the malignant peripheral nerve sheet tumor involving the brachial plexus
		Brinkman M et al., 2018 [20]	Evaluation of the most commonly used (semi-)quantitative parameters of 18F-FDG PET/CT to detect malignant transformation of neurofibromas in neurofibromatosis type 1
		Yadav D et al., 2020 [21]	Role of 18F-FDG PET/computed tomography in prognostication and management of malignant peripheral nerve sheath tumors
		Yun JS et al., 2021 [2]	Peripheral nerve sheath tumor: differentiation of malignant from benign tumors with conventional and diffusion-weighted MRI
		Nishida Y et al., 2021 [22]	Limitations and benefits of FDG-PET/CT in NF1 patients with nerve sheath tumors: A cross-sectional/longitudinal study
		Liu J et al., 2022 [23]	Image-Based Differentiation of Benign and Malignant Peripheral Nerve Sheath Tumors in Neurofibromatosis Type 1
		Ristow I et al., 2022 [24]	Evaluation of magnetic resonance imaging-based radiomics characteristics for differentiation of benign and malignant peripheral nerve sheath tumors in neurofibromatosis type 1
		Koike H et al., 2022 [25]	Diffusion-Weighted Magnetic Resonance Imaging Improves the Accuracy of Differentiation of Benign from Malignant Peripheral Nerve Sheath Tumors
		Debs P et al., 2022 [26]	MR Neurography of Peripheral Nerve Tumors and Tumor-Mimics
Risk factors/genetic disorders	5	Ferner RE et al., 2002 [27]	International consensus statement on malignant peripheral nerve sheath tumors in neurofibromatosis
		Reilly KM et al., 2017 [28]	Neurofibromatosis Type 1-Associated MPNST State of the Science: Outlining a Research Agenda for the Future
		Zipfel J et al., 2020 [29]	Surgical management of peripheral nerve sheath tumours in children, with special consideration of neurofibromatoses
		Pulliam S et al., 2022 [30]	Metastatic Malignant Peripheral Nerve Sheath Tumor (MPNST) in Neurofibromatosis Type 1: Challenges in Diagnosis and Management
		Ejerskov C et al., 2023 [31]	Clinical Characteristics and Management of Children and Adults with Neurofibromatosis Type 1 and Plexiform Neurofibromas in Denmark: A Nationwide Study
Preoperative biopsy	5	Brahmi M et al., 2015 [32]	Diagnostic Accuracy of PET/CT-Guided Percutaneous Biopsies for Malignant Peripheral Nerve Sheath Tumors in Neurofibromatosis Type 1 Patients
		Mito JK et al., 2017 [33]	Role of Histone H3K27 Trimethylation Loss as a Marker for Malignant Peripheral Nerve Sheath Tumor in Fine-Needle Aspiration and Small Biopsy Specimens
		Graham DS et al., 2019 [34]	Oncologic Accuracy of Image-guided Percutaneous Core-Needle Biopsy of Peripheral Nerve Sheath Tumors at a High-volume Sarcoma Center
		Pendleton C et al., 2021 [35]	Percutaneous image-guided biopsy in malignant peripheral nerve sheath tumors
		Yanagisawa K et al., 2022 [36]	Successful Radiotherapy of Primary Malignant Peripheral Nerve Sheath Tumor of the Lung
Radiotherapy	3	Kahn J et al., 2014 [37]	Radiation therapy in management of sporadic and neurofibromatosis type 1-associated malignant peripheral nerve sheath tumors
		Vitolo V et al., 2019 [38]	Carbon Ion Radiotherapy in the Management of the Tumors of the Peripheral Nervous System
		Roohani S et al., 2023 [39]	The role of radiotherapy in the management of malignant peripheral nerve sheath tumors: a single-center retrospective cohort study
Clinical management	18	Ferrari A et al., 2007 [8]	Management of childhood malignant peripheral nerve sheath tumor
		Minovi A et al., 2007 [40]	Malignant peripheral nerve sheath tumors of the head and neck: management of 10 cases and literature review
		Lin CT et al., 2009 [41]	Treatment of a malignant peripheral nerve sheath tumor
		Stucky CC et al., 2012 [9]	Malignant peripheral nerve sheath tumors (MPNST): the Mayo Clinic experience
		Tu A et al., 2014 [42]	MPNST after Radiosurgery: A Report and Review of the Literature
		Thway K et al., 2014 [4]	Malignant peripheral nerve sheath tumor: pathology and genetics
		James AW et al., 2016 [7]	Malignant Peripheral Nerve Sheath Tumor
		Valentin T et al., 2016 [6]	Management and prognosis of malignant peripheral nerve sheath tumors: The experience of the French Sarcoma Group (GSF-GETO)
		Kim A et al., 2017 [43]	Malignant Peripheral Nerve Sheath Tumors State of the Science: Leveraging Clinical and Biological Insights into Effective Therapies
		Guha D et al., 2018 [5]	Management of peripheral nerve sheath tumors: 17 years of experience at Toronto Western Hospital
		Rais G et al., 2020 [44]	Successful Management of Intrathoracic Phrenic Malignant Peripheral Nerve Sheath Tumor by Multimodal Treatment
		Marickar YMF et al., 2020 [45]	Malignant peripheral nerve sheath tumour—A long story: Case report
		Hassan A et al., 2021 [46]	Systemic Options for Malignant Peripheral Nerve Sheath Tumors
		Gaba S et al., 2021 [47]	Clinical Outcomes of Surgical Management of Primary Brachial Plexus Tumors
		González-Muñoz T et al., 2022 [48]	The Need for New Treatments Targeting MPNST: The Potential of Strategies Combining MEK Inhibitors with Antiangiogenic Agents
		Knight SWE et al., 2022 [49]	Malignant Peripheral Nerve Sheath Tumors-A Comprehensive Review of Pathophysiology, Diagnosis, and Multidisciplinary Management
		Yao C et al., 2023 [50]	Malignant Peripheral Nerve Sheath Tumors: Latest Concepts in Disease Pathogenesis and Clinical Management
		Vetrano IG et al., 2023 [51]	Editorial: Improving our understanding of the management and pathogenesis of rare and neglected tumours of the central and peripheral nervous system

**Table 2:** Distribution of the different histopathological diagnoses of peripheral nerve neoplasms operated on between 2018 and 2024 included in the patient cohort used to validate the algorithm. MPNSTs accounted for 4.35% of all lesions (8.3% of the lesions meeting the inclusion criteria) in our series.

Histological Diagnosis	N° Neoplasm	Percentage (%)
Schwannoma/ancient schwannoma	29/36	80.5%
Ganglioneuroma	1/36	2.8%
Fibromatosis-desmoid	1/36	2.8%
Glomus tumour	1/36	2.8%
Haemangioma	1/36	2.8%
MPNST	3/36	8.3%

**Table 3:** A brief summary of the three patients affected by MPNSTs. GTR: Gross Total Resection; STR: Subtotal Resection; NA: Not Applicable.

ID	Gender	Age (Years)	Location	Neoadjuvant Therapy	Surgery	Surgical Margin	Radiotherapy	Chemotherapy
Case 1	M	49	Right brachial plexus	NA	GTR	R0	Yes	No
Case 2	F	46	Left brachial plexus	NA	STR	R2	Yes	Yes
Case 3	F	51	Left lumbar plexus	Radiotherapy	GTR	R0	Yes	No

## Data Availability

Due to patient privacy concerns, the data presented in this study are available from the corresponding author upon reasonable request.

## Abbreviations

The following abbreviations are used in this manuscript:

PRISMA	Preferred Reporting Items for Systematic Reviews and Meta-Analyses
PNT	Peripheral Nerve Tumours
BPNSTs	Benign Peripheral Nerve Sheath Tumours
MPNSTs	Malignant Peripheral Nerve Sheath Tumours
NF	Neurofibromatosis
EoM	Evidence of Malignancy
WHO	World Health Organization
GTR	Gross Total Resection
STR	Sub Total Resection
MRC	Medical Research Council
ADC	Apparent Diffusion Coefficient
MRI	Magnetic Resonance Imagining
PET/CT	Positron Emission Tomography/Computed Tomography
US	Ultrasound
ECO	Echography
DWI	Diffusion-Weighted Imaging
PN	Plexiform Neurofibroma
^18^FDG	18F-labeled fluorodeoxyglucose
SUV	Standard Uptake Value
T/L	Tumour to Liver ratio
TSE	Turbo Spin Echo
STIR	Short Tau Inversion Recovery
